# Marine Brown Algae-Derived Compounds as Potential Inhibitors of Japanese Encephalitis Virus RNA-Dependent RNA Polymerase

**DOI:** 10.3390/md22020092

**Published:** 2024-02-17

**Authors:** Saud O. Alshammari

**Affiliations:** Department of Pharmacognosy and Alternative Medicine, Faculty of Pharmacy, Northern Border University, Rafha 76321, Saudi Arabia; saud.o.alshammari@nbu.edu.sa; Tel.: +966-551055990

**Keywords:** Japanese encephalitis, RdRp, Phaeophyceae, brown algae, molecular dynamics

## Abstract

The Japanese encephalitis virus (JEV) is a mosquito-borne flavivirus that primarily affects people in Asia and seriously threatens public health. Considering the rising occurrence rates and lack of targeted antiviral treatments, it is essential to comprehend and tackle obstacles related to JEV in order to lessen its influence on world health. This investigation explores compounds derived from marine brown algae (Phaeophyceae) as potential inhibitors of JEV RNA-dependent RNA polymerase (RdRp), a critical enzyme in the virus’s replication cycle. Employing the computational virtual screen approach, four compounds, i.e., CMNPD16749, CMNPD2606, CMNPD27817, and CMNPD23662, with favorable binding energies ranging from −15.7 Kcal/mol to −13.9 kcal/mol were identified. Subsequently, through molecular docking analysis, the interactions responsible for the binding stability between the target protein and hit molecules compared to the reference molecule Galidesvir were studied. Further, through extensive molecular dynamic (MD) simulation studies at 200 ns, it was confirmed that each docked complex showed acceptable dynamic stability compared to the reference molecule. These findings were further validated using MM/PBSA free binding energy calculations, PCA analysis and free energy landscape construction. These computational findings suggested that the brown algae-derived compounds may act as an antiviral drug against JEV infection and lay a crucial foundation for future experimental studies against JEV.

## 1. Introduction

The quest for effective antiviral agents against the Japanese encephalitis virus (JEV) remains a significant challenge in the realm of infectious diseases. JEV, a member of the Flavivirus genus, is the primary cause of viral encephalitis in human populations across Southeast Asia and the Western Pacific. Mosquitoes vector the disease and manifest a broad spectrum of clinical symptoms, from mild fever to severe neurological disorders, including encephalitis, which may lead to death or permanent disability [1]. An estimated 68,000 clinical cases occur annually, emphasizing the urgent need for therapeutic interventions [2,3]. RNA-dependent RNA polymerase (RdRp), a critical enzyme in the replication cycle of JEV, has emerged as a promising target for antiviral drug discovery. RdRp catalyzes the synthesis of the viral RNA genome. Thus, its inhibition could potentially arrest viral replication [4,5]. Given that JEV shares a degree of genetic and structural homology with other medically important flaviviruses, RdRp inhibitors could have broad-spectrum antiviral effects, a valuable trait in antiviral drug development [6]. Historically, marine organisms have been a rich source of bioactive compounds, with a notable track record in drug discovery [7]. Marine-derived molecules possess unique chemical structures not found in terrestrial compounds, which have been translated into therapeutic agents for a myriad of conditions, including viral infections [8,9,10,11]. Their diverse secondary metabolites, produced as chemical defenses against the complex marine environment, have provided novel scaffolds for developing antiviral agents [9].

Recent research has revealed that five FDA-approved drugs like manidipine, benidipine hydrochloride, nelfinavir mesylate, butoconazole nitrate, and mitotane show promising antiviral activity against JEV [12]. Additionally, earlier studies have emphasized the effectiveness of nucleoside analogs in selectively targeting both Dengue fever and JEV [13]. However, while all these compounds demonstrate antiviral potential, they are not target-specific molecules. Therefore, identifying molecules specific to their targets remains a crucial area for further investigation in the treatment and management of these viral infections.

Computational drug discovery, encompassing virtual screening and molecular docking investigations, has evolved into an essential tool for pinpointing potential inhibitors targeting therapeutic markers. This methodology facilitates the swift and cost-efficient assessment of extensive compound libraries against particular protein targets [14]. Integrating computational methods with the wealth of chemical diversity offered by marine compounds presents a promising avenue for identifying novel inhibitors against JEV RdRp [15].

The present manuscript aims to bridge the gap between marine bioactive compounds (brown algae) and antiviral therapy for JEV by identifying potential inhibitors of the virus’s RdRp through computational drug discovery. By employing computation drug discovery tools, the research evaluates brown algae-derived compounds’ binding affinity and stability to the RdRp active site [16]. The Pheophyceae class of algae, also known as brown algae, is a large and diverse group of marine organisms that range from simple filaments to complex seaweeds [17,18]. Their golden-brown color comes from the pigment fucoxanthin and the polyphenolic compounds called pheophycean tannins, which help them cope with environmental stress and protect them from harmful radiation [17]. One of the benefits of brown algae is that they produce a variety of substances that positively affect human health and other applications. One of these substances is phlorotannin, which is a particular type of secondary metabolite that is only found in brown algae. Phlorotannin has many different biological activities and can be used in various fields. Secondary metabolites are compounds that are not essential for the algae’s survival but are involved in their interactions with other organisms and their adaptation to their habitat. These substances are abundant in marine algae and can support their defenses against infections. In addition to phlorotannin, phytosterol and polyphenol are two significant secondary metabolites found in brown algae with a wide range of compounds essential to numerous biological processes [18,19]. Identifying lead compounds with high binding affinities offers a starting point for developing new antiviral drugs against JEV, potentially with broad-spectrum activity against related flaviviruses.

## 2. Results

### 2.1. Virtual Screening Analysis

Virtual screening serves as a critical computational technique in identifying potential bioactive compounds from large libraries, targeting specific biological interactions. This research focused on marine brown algae, leading to the identification of four significant natural compounds through virtual screening. The process involved evaluating 1212 compounds from marine brown algae using Lipinski’s rule of five. Ultimately, 153 compounds were obtained, which displayed binding scores between −15.7 kcal/mol and −6.3 kcal/mol (detailed in Supplementary Table S1). Four compounds stood out due to their high binding scores and strong molecular interactions: CMNPD16749, CMNPD2606, CMNPD27817, and CMNPD23662 (Table 1). These were selected for further examination. 

Brown algae, particularly those from marine sources, are increasingly recognized for their potential as sources of antiviral agents. This is primarily due to their unique structural and biological characteristics. The efficacy of marine brown algae in this context has been supported by several academic studies, including the work by Mayer and colleagues in 2013 [8].

### 2.2. Re-Docking and Intermolecular Interaction Studies

Re-docking plays a crucial role in drug development, entailing ligand molecule re-alignment with a receptor molecule. This step is vital for evaluating the precision of docking and the essential interactions between the ligand and the receptor’s binding site. In this study, selected ligands and a reference molecule underwent re-docking with the JEV RdRp protein. This analysis generated at least 10 poses per complex, with the pose showing the highest docking energy selected for further investigation of complex stability and ligand–protein binding affinity.

This research’s virtual screening phase identified four promising natural compounds from marine brown algae. These compounds were chosen based on their binding energy during the screening, ranging from −15.7 Kcal/mol to −6.3 Kcal/mol. The four natural compounds, RdRp-CMNPD16749(−15.7 Kcal/mol), RdRp-CMNPD2606 (−14.8 Kcal/mol), RdRp-CMNPD27817 (−14.4 Kcal/mol), and RdRp-CMNPD23662 (−13.9 Kcal/mol), along with the reference molecule Galidesivir, which had the −8.1 Kcal/mol binding energy, were earmarked for deeper analysis. Galidesivir is a broad-spectrum antiviral drug, effective against several RNA viruses, including families like Flaviviridae, Coronaviridae, and Filoviridae [24,25,26,27,28,29,30]. It was chosen as a reference ligand for this study due to its proven efficacy against Flaviviruses, its mechanism of action, and the valuable structural and pharmacological insights it provides.

In the re-docking process, the reference ligand was removed from its binding site and reattached to the same area on the target protein, resulting in a re-docking score of −8.1 kcal/mol. Previous research has highlighted the strong binding affinity of natural compounds with JEV RdRp. For instance, Dwivedi and his co-workers discovered natural inhibitors from Azadirachta indica against RdRp, with compounds like Gedunin, Nimbolide, Ochinin acetate, and Kulactone showing docking scores ranging from −10.4 to −11.0 kcal/mol [31]. Similarly, Yadav and his team identified potent phytocompounds such as Echinacoside and Kaempferol-3-glucoside from *Echinacea augustifolia*, chosen for their high negative docking scores [32]. These studies confirm that natural compounds have the substantial potential to be promising inhibitors of RdRp protein.

**Figure 1:**
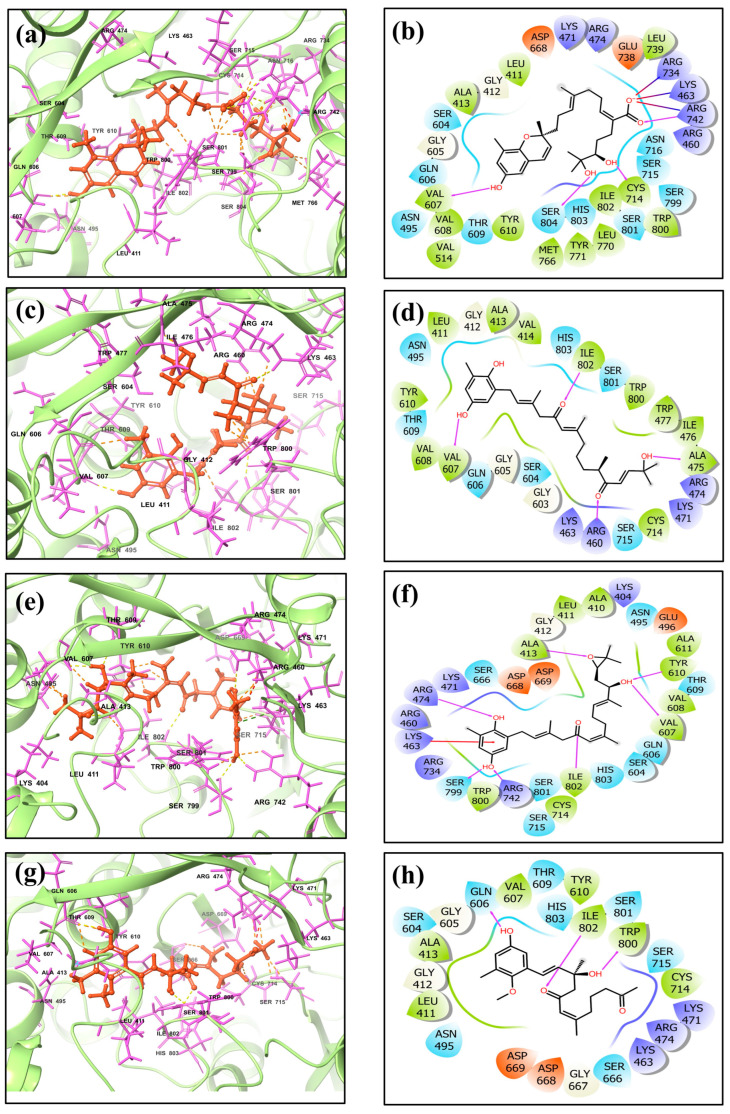
Diagram of the 3D and 2D protein–ligand interaction of JEV–RdRp with selected brown alga natural compounds: (**a**,**b**) CMNPD16749, (**c**,**d**) CMNPD2606, (**e**,**f**) CMNPD27817, and (**g**,**h**) CMNPD23662 complexes. These images were generated using free academic maestro [34].

The functionality, stability, and selectivity of a complex consisting of a protein and ligand are largely determined by its intramolecular interactions. The complex’s total binding affinity and, by extension, its biological activity are greatly influenced by these interactions. Since intramolecular interactions affect the effectiveness and selectivity of possible treatments, understanding and improving them is crucial to drug discovery and design processes [33]. In the JEV–RdRp–CMNPD16749 complex, four hydrogen bonds were identified between residues Val^607^, Cys^714^, Arg^742^, and Ser^804^, indicating a stable interaction between the compound CMNPD16749 and the RdRp protein. Similarly, the docking interactions of JEV–RdRp–CMNPD2606 involved four hydrogen bonds with residues Ala^475^, Arg^460^, Val^607^, and Ile^802^, suggesting a strong binding affinity. The JEV–RdRp–CMNPD27817 complex exhibited seven hydrogen bonds with Ala^413^, Arg^474^, Val^607^, Tyr^610^, Arg^742^, Ser^799^, and Ile^802^ residues, as well as a pi-cation interaction in the Lys^463^ residue, indicating diverse yet robust interactions. In the RDRP–CMNPD23662 complex, three hydrogen bonds were identified with Gln^606^, Trp^800^, and Ile^802^ residues. In contrast, the JEV–RdRp–galidesivir complex formed hydrogen bonds with Pro^464^, Arg^734^, Gly^735^, and Glu^738^ residues, highlighting specific binding patterns for the reference molecule galidesivir. The molecular interaction analysis displayed that the JEV–RdRp–CMNPD27817 complex exhibited the maximum number of bond formations compared to the reference complex. The JEV–RdRp–CMNPD16749 and JEV–RdRp–CMNPD27817 complexes exhibited the same number of hydrogen bonds as the reference complex. These detailed molecular interactions provide insights into the binding modes of the compounds during molecular docking with RdRp, emphasizing the potential for these interactions to influence the inhibitory activity against JEV–RdRp. The complexes were further illustrated in 3D and 2D interactions, which were further studied using molecular dynamic simulation (Figure 1, Table 2, and Supplementary Figure S1).

### 2.3. Molecular Dynamic Simulation Studies

This segment explores the findings from molecular dynamics (MD) simulations carried out over 200 nanoseconds. The focus was on examining the stability and adaptability of the top five complexes previously identified. The key metrics observed during the MD simulations included the RMSD for both the protein and the ligand, along with the RMSF of the protein. These metrics are essential as they provide insight into the evolution and adaptability of the protein–ligand complexes, revealing their structural dynamics and variations over time.

Additionally, the research involved a comprehensive examination of the formation of hydrogen bonds throughout the 200 ns period. This aspect is vital for grasping the interactive behavior between the protein and ligand within the complexes. By examining the nature and strength of these hydrogen bonds, the study provided essential insights into the binding stability of the complexes. It offered clues about their potential mechanisms of action. This comprehensive analysis contributes significantly to our understanding of the behavior and efficacy of these molecular interactions.

#### 2.3.1. RMSD and RMSF Analysis

This research used an MD simulation to explore the dynamic interactions and structural characteristics of four naturally occurring compounds when they engage with the JEV virus RdRp protein. The primary goal of this simulation was to evaluate the stability and interaction patterns, as well as the conformational changes within the protein-ligand complexes.

**Figure 2:**
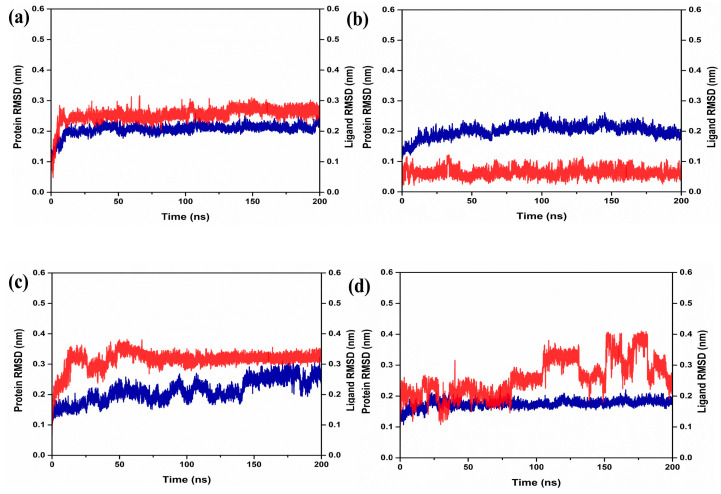
RMSD graph generated from molecular dynamics simulation over 200 ns of the selected docked complex that is (**a**) RdRp–CMNPD16749, (**b**) RdRp–CMNPD2606, (**c**) RdRp–CMNPD27817, and (**d**) RdRp–CMNPD23662. Here, the blue color in the RMSD graph represents the protein RMSD, and the red color represents the ligand RMSD or natural compound’s RMSD in each RdRp–natural compound complex.

A critical component of the analysis was the RMSD, which was utilized to evaluate the stability of these complexes throughout the 200 ns simulation. Notably, the RdRp protein, when combined with the docked natural compounds and the reference complex, displayed dynamic stability, with RMSD values remaining below 0.30 nm (Figure 2). Specifically, the compound RdRp–CMNPD16749 exhibited a consistently stable RMSD of 0.3 nm or less during the simulation period. The ligand molecule of RdRp–CMNPD2606 also showed remarkable stability, maintaining an RMSD of less than 0.1 nm, indicating its significant stability while bound to the target protein.

RdRp–CMNPD27817 presented minor fluctuations (around 0.3 nm) in the initial stages of the simulation but reached a state of equilibrium after about 50 ns, revealing conformational shifts in the protein structure post-binding. RdRp–CMNPD23662 demonstrated stable protein RMSD values, with minor ligand RMSD fluctuations ranging from 0.2 to 0.4 nm, particularly after 100 ns of simulation. The RdRp–galidesivir consistently exhibited very low RMSD values (between 0.2 to 0.4 nm) until the end of the 200 ns simulation, suggesting its stability. The results from the RMSD assessment revealed that each of the compounds demonstrated a consistent level of stability, suggesting their potential to effectively inhibit the target protein in a manner similar to that of the reference complex RdRp–galidesivir.

**Figure 3:**
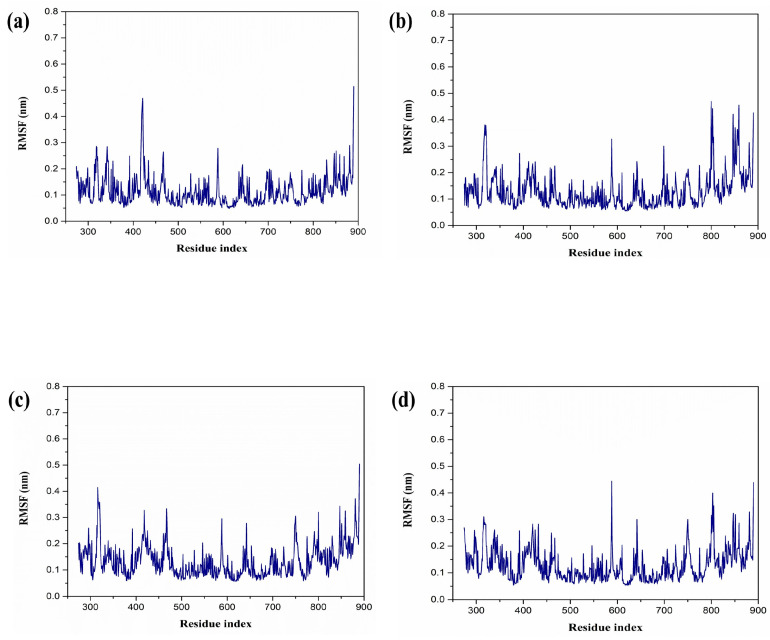
RMSF graph generated from molecular dynamics simulation over 200 ns (**a**) RdRp–CMNPD16749, (**b**) RdRp–CMNPD2606, (**c**) RdRp–CMNPD27817, and (**d**) RdRp–CMNPD23662. Here, the blue color represents the protein RMSF in each complex.

Moreover, the study employed RMSF to quantify the average variations in atomic positions relative to their average coordinates throughout the simulation. Generally, elevated RMSF values indicate greater flexibility and variability, while lower values are associated with increased structural rigidity and stability. In this specific analysis, the RdRp protein exhibited a notably low RMSF value (below 0.5 nanometers), with only minor residual fluctuations in all the selected complexes (Figure 3). Similar variations were observed in the reference complexes, as well as RdRp–galidesivir. This finding aligns with the stability of the protein–ligand complexes, as indicated in the RMSD assessment.

#### 2.3.2. Hydrogen Bond Analysis

**Figure 4:**
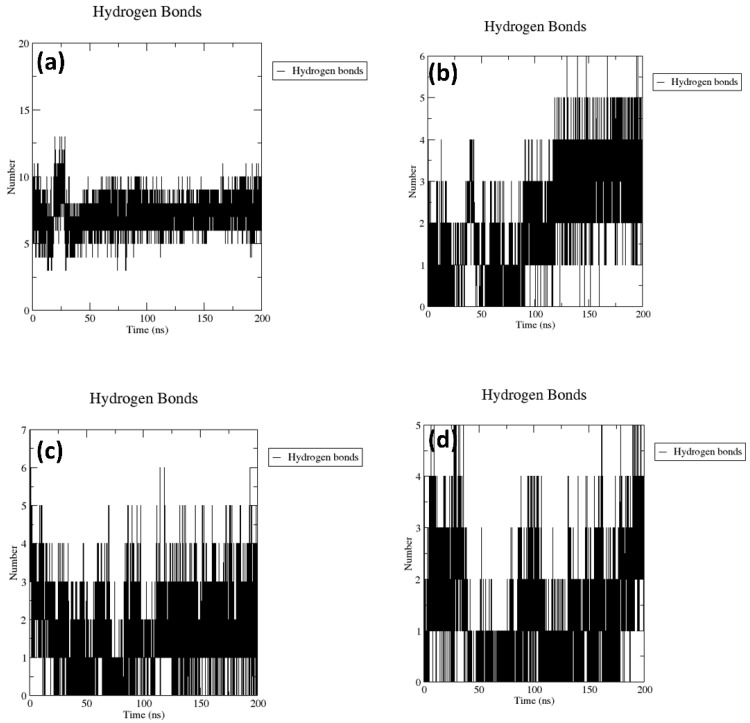
The number of hydrogen bonds present in the JEV–RdRp docked with brown alga natural compounds, i.e., (**a**) RdRp–CMNPD16749, (**b**) RdRp–CMNPD2606, (**c**) RdRp–CMNPD27817, and (**d**) RdRp–CMNPD23662 during the 200 ns MD simulation.

In order to evaluate the binding stability of the compound post-molecular dynamics (MD) simulation, the formation of hydrogen bonds was quantified. The analysis revealed that the RdRp–CMNPD16749 complex formed the highest number of hydrogen bonds, reaching a total of 10 throughout the 200 ns simulation period. In contrast, the other complexes typically formed between four and five hydrogen bonds during the same timeframe. The reference complex, for comparison, maintained between five and eight hydrogen bonds until the conclusion of the simulation. These results led to the inference that the RdRp–CMNPD16749 complex demonstrated superior stability relative to both the reference complex and other protein–ligand complexes under study, as evidenced by its higher count of hydrogen bonds (refer to Figure 4 and Supplementary Figure S4 for details).

### 2.4. MM/PBSA Calculation

The MM/PBSA calculation is a critical tool for evaluating the binding free energy of protein–ligand complexes. It combines molecular mechanics (MM) energies with solvation energies derived from the Poisson–Boltzmann surface area (PBSA) method. This approach offers insights into the energetics of molecular interactions, aiding in understanding the stability and affinity of the complexes. According to the results, RdRp–CMNPD16749 displayed a total of −63.43 kcal/mol. The RdRp–CMNPD2606 complex showed an ΔG of −33.45 kcal/mol. The ΔG value for the RdRp–CMNPD27817 complex was −36.53 kcal/mol. Similarly, the binding free energy (ΔG) for the RdRp–CMNPD23662 complex was recorded at −23.54 kcal/mol. This is compared to the reference complex’s ΔG, which was measured at −33.06 kcal/mol (see Supplementary Table S2). Notably, the ΔG values of the complexes being studied were closely aligned with that of the reference, reinforcing the notion of their stable interaction with the target protein. This observation is further supported by the firm and efficient binding demonstrated between the compounds and the RdRp protein, as evidenced by their similarity to the reference complex in terms of ΔG values. Their insignificant standard deviations further support the estimated binding energies’ reliability and uniformity. The overall results of these studies suggest that the compounds under investigation have a substantial binding affinity for the target protein, indicating that these compounds may be persistent and efficient ligands that inhibit their mechanism of action.

### 2.5. Principal Component Analysis

**Figure 5:**
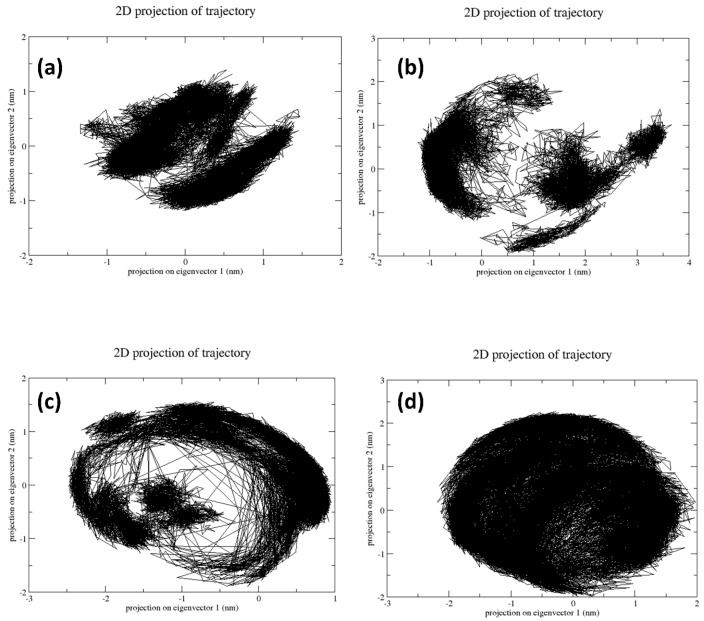
PCA graph generated after molecular dynamics simulation over 200 ns: (**a**) RdRp–CMNPD16749, (**b**) RdRp–CMNPD2606, (**c**) RdRp–CMNPD27817, and (**d**) RdRp–CMNPD23662.

The principal component analysis focuses on the dimensionality reduction of multidimensional data without losing significant information (Figure 5). The scatter plots of the respective complex generated from their simulation trajectories help analyze the dynamic stability of the complex. Herein, in the case of the RdRp–CMNPD16749 complex, the scatter plot appears to be in a dense form. This depicts that the complex does not undergo any conformational changes, and common conformers are present during the MD simulation. This states that the complex is in a dynamic, stable state. In the case of the RdRp–CMNPD2606 complex, the plots are less dense and scattered in nature. It describes that the complex has undergone certain conformational changes during the simulation. Similarly, the scatter plot of the RdRp–CMNPD16749 complex was also less dense and scattered. This also states that this complex has also undergone minor conformational changes. However, the RdRp–CMNPD23662 complex showed a dense plot, denoting the absence of any conformational changes and stability. The genidesivir compound dense plot has minor scattering, indicating the presence of insignificant conformational changes with acceptable stability.

### 2.6. Free Energy Landscape

In the framework of molecular dynamics simulations, a free energy landscape offers a thorough understanding of a biomolecular system’s energy distribution and conformational dynamics. The representation is multi-dimensional and shows the different states and energy levels that the system can access over time. A free energy landscape can be used to see the various conformations and energy minima that a protein–ligand complex can take on during its dynamic function [35].

In essence, the landscape allows scientists to locate energy barriers, stable states, and transitions between various conformations. In a landscape, stable states and energy barriers are represented by valleys and peaks, respectively [36]. To better understand the interactions, conformational shifts, and overall stability of a biomolecular complex, it is crucial to understand its thermodynamics and kinetics. This understanding is often facilitated by exploring the free energy landscape of the system. Researchers commonly use techniques like principal component analysis (PCA) to simplify the complexity of data by reducing its dimensions. This simplification allows for a more accessible and insightful representation of the free energy landscape [37]. 

**Figure 6:**
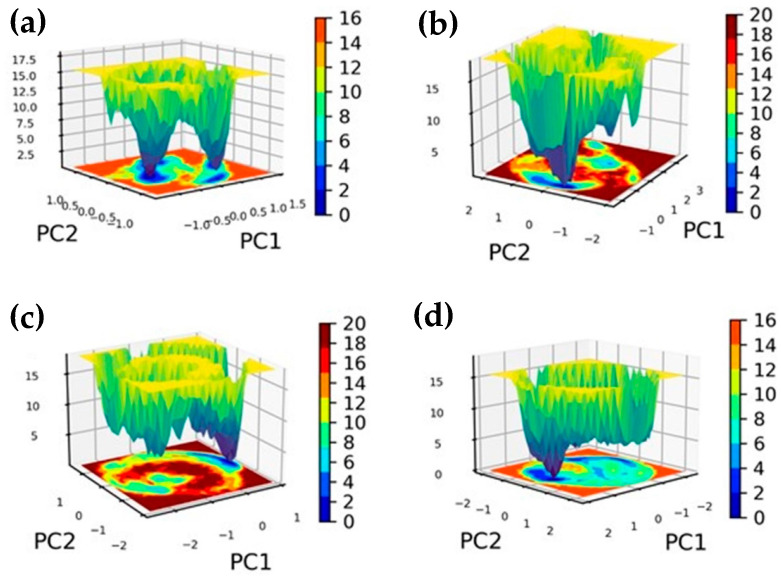
This graph illustrates the free energy landscape of the JEV-RdRp complex docked with brown algae natural compounds, namely: (**a**) RdRp–CMNPD16749, (**b**) RdRp–CMNPD2606, (**c**) RdRp–CMNPD27817, and (**d**) RdRp–CMNPD23662.

For this study, a three-dimensional (3D) free energy landscape was constructed using the first (PC1) and second (PC2) principal components obtained from PCA. These 3D graphs formed the basis of the free energy landscapes for both the ligand-bound complexes and a reference control group (Figure 6 and Supplementary Figure S4).

These graphical representations are pivotal in shedding light on the energy dynamics associated with conformational changes within the complexes. They offer a visual interpretation of how the conformations evolve over the course of the simulation. Notably, the 3D projections effectively display the dynamic conformational alterations, forming a low-energy structure. This structure is visually distinctive, resembling a narrow, funnel-like shape, highlighting the energy efficiency and stability of the final conformation achieved by the complexes [38]. 

The examination of the FEL also revealed areas with deep blue hues, indicating localized energy troughs. These troughs signify that during specific phases of the simulation, the protein configurations reached states of optimal energy efficiency. Dark blue zones within the complexes signify their attainment of configurations with minimal energy levels.

Within the broader context, terms like “narrow basin” and “wide basin” describe different energy states that molecules can achieve. For example, in Figure 6, complexes RdRp–CMNPD16749, RdRp–CMNPD23662, and the reference molecule displayed narrow basins, suggesting highly stable states with few transitions between different states. In contrast, RdRp–CMNPD2606 and RdRp–CMNPD27817 exhibited wider basins, indicating various accessible states and more frequent transitions.

Specifically, RdRp–CMNPD16749 was notable for its narrow basins, signifying deep energy wells and limited state transitions. In contrast, RdRp–CMNPD27817 and RdRp–CMNPD23662 demonstrated a broader range of conformational states.

Structures corresponding to minimum, intermediate, and maximum energy levels were analyzed further to understand these complexes’ interactions and conformational changes. This approach provided valuable insights into molecular dynamics, revealing varied conformational states and interaction patterns indicative of a stable binding arrangement.

Notably, the minimum energy state was consistently observed at 0 kJ/mol for all complexes, while the relative maximum energy states fell within the range of 16 kJ/mol to 20 kJ/mol. This quantitative perspective offers a clear understanding of the energy landscape of these molecular systems.

Lastly, the free energy landscape of the reference molecule exhibited two conformations with similar Gibbs free energy levels, suggesting a potential decrease in stability. RdRp–CMNPD23662’s landscape featured a primary global minimum along with a secondary local minimum with slightly lower Gibbs free energy, indicating a possible alternate, less stable conformation. RdRp–CMNPD2606’s landscape revealed a single global minimum, implying the presence of one stable conformation.

## 3. Discussion

In this study, through structure-based virtual screening, four natural compounds, i.e., CMNPD16749, CMNPD2606, CMNPD27817, and CMNPD23662, derived from marine brown algae were selected for the in-depth studies. These compounds showed the highest negative binding score during the virtual screening analysis, which is considered one of the most significant compound selection criteria of the virtual screening analysis [39]. Virtual screening has been a prominent technique in identifying potential drug molecules. This technique has been a significant contributor to identifying various antiviral compounds, especially for treating COVID-19 [40]. For example, Prasertsuk and team identified five major proteins from the hemp seed trypsinized peptidome against 3 Clpro proteins of SARS-CoV-2 using computational screening [41]. Similarly, a virtual screening technique was also used to identify the binding affinity of the guanidine alkaloids against various target proteins of SARS-CoV-2, such as Mpro, spike glycoprotein, etc. Interestingly, the binding affinities of all these compounds were significant and showed a maximum range of up to −9.06 Kcal/mol [42]. Another study used this technique to identify an inhibitory peptide from the putative hydrolyzed peptidome of Rice Bran against the Mpro protein of SARS-CoV-2 [43]. These findings confirmed that virtual careening can be a significant contributor to identifying promising inhibitory molecules from various sources. Further, the re-docking of these selected compounds with the target protein showed prominent interactions such as hydrogen and hydrophobic bonds. These bonds are crucial in increasing the binding affinity and overall stability of the docked complex [44]. Herein, the CMNPD27817 compound exhibited the maximum number of hydrogen bonds compared to all other selected compounds and the reference compounds, stating the fact that this compound possesses maximum binding affinity due to a higher number of interactions, primarily due to the presence of the maximum number of hydrogen bonds [45]. The CMNPD23662 compound exhibited the minimum number of hydrogen bonds compared to all the compounds and reference compounds. The other two compounds and the reference compound displayed the same amount of hydrogen bond formation. Based on the observation of the interactions formed during the re-docking analysis. All the compounds showed an acceptable binding affinity with the target protein compared to the reference molecule galidesivir and other reported inhibitors.

The molecular dynamics simulation analysis further validated the stability of the selected complex. The protein RMSD of each complex exhibited low RMSD. This scenario was observed during the literature studies, which confirms that the protein showed dynamic stability during the simulation. Interestingly, the RMSD analysis of the selected compound has remarkable dynamic stability. A similar situation was observed with compounds obtained from *Azadicrachta indica*, and *Echinacea angustifolia* docked with JEV–RdRp protein [31,32]. These findings confirm that the ligand molecules are also in a stable conformational state during the simulation. Altogether, the protein-ligand RMSD analysis of the selected complex, when compared with the reference complex, displayed remarkable stability, and based on the RMSD analysis from previous literature, it can be stated that these compounds have dynamic stability and may be promising inhibitory molecules against JEV–RdRp protein. Furthermore, the residues of the protein in each complex showed minimum fluctuation. This also confirms the stability of the protein during the binding of the selected ligand molecules. The maximum number of hydrogen bonds was seen in the RdRp protein. 

The free binding analysis using the MM/PBSA calculation proved that the CMNPD16749 compound exhibited the highest binding free energy. This confirms that this compound has the highest stability when docked with JEV–RdRp protein compared to other natural and reference compounds. To further validate the dynamic stability of these complexes, the utilization of principal component analysis and the free energy landscape of the plot was analyzed. CMNPD16749 and CMNPD23662 showed dense plots and less conformational changes than CMNPD2606, CMNPD27817, and the reference complex.

Based on the computational experiment, we identified four promising natural compounds, specifically CMNPD16749, CMNPD2606, CMNPD27817, and CMNPD23662, showing acceptable inhibitory properties against the RdRp protein. These compounds have not been previously examined for their impact on RdRp or their ability to combat different types of viral encephalitis. The absence of previous research, whether conducted in a laboratory setting, in living organisms, or using computer-based approaches, emphasizes the originality and importance of the discoveries provided in this work. This study will also be a reference for other researchers aiming to discover new antiviral substances derived from natural sources, emphasizing reducing toxicity and enhancing effectiveness within a shorter period. This will be valuable for future efforts in treating viral infections.

## 4. Materials and Methods

### 4.1. Virtual Screening

**Figure 7:**
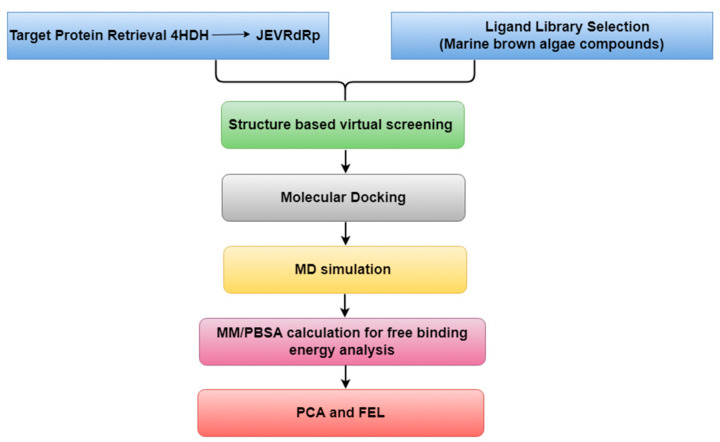
The workflow of the experiment performed during this research.

As represented in Figure 7, the virtual screening analysis was performed using the MTi-OpenScreen web-based platform [46]. The ligand library, which consists of natural compounds derived from marine brown algae, was collected from the Comprehensive Marine Natural Products Database (https://www.cmnpd.org/, accessed on 14 October 2023) [47]. The protein structure for virtual screening was retrieved from the Protein Data Bank (PDB ID: 4HDH) [5,48]. The protein structure contains ATP molecules in their binding site. Hence, the ATP binding was coordinated, i.e., *X*: −34.66, *Y*: 9.67 and *Z*: −28.19, were obtained to build a grid box of size 30 × 30 × 30 Å. The ligand library was followed by being docked into the same binding site during the screening process after removing the ATP molecule from the target protein. The standard docking protocol was used during the screening, and the compounds that exhibited maximum negative docking scores were selected for further analysis of their binding affinities and interaction profiling with the active site residues of the RdRp protein.

### 4.2. Re-Docking

To validate the docking poses obtained from the virtual screening, re-docking was performed in the same grid box using AutoDock Vina integrated within the UCSF Chimera environment [49,50]. Moreover, galidesivir, a broad-spectrum antiviral drug molecule, was also docked within the same binding site, and the docked RdRp–galidesivir complex was considered as the reference complex for the comparative study with the selected docked RdRp–natural complex. The re-docking process confirms the reproducibility and reliability of the virtual screening results by reducing the chances of choosing false positives. The best conformations were subjected to a consensus scoring scheme and visually inspected for their binding affinity and intermolecular interaction consistency. 

### 4.3. Molecular Dynamics Simulation

The Gromacs molecular dynamic (MD) simulation package was utilized to perform the 200 ns simulation of the selected RdRp–natural compound complex and the reference complex, RdRp–galdisivir [51]. The complexes were prepared by removing the ions, and hydrogen atoms were added to the complexes. The CHARMM36 force field was used during the simulation, and the complexes’ topology was generated using the pdb2gmx tool present in GROMACS software [52]. A cubic simulation box was created, and the complexes were placed into it. The maximum amount of space was provided in the box to avoid periodic boundary conditions. Then, the TIP3P water module was added to the complex containing the box by maintaining a buffer distance of 1 nm from the boundary of the box. This was performed for system solvation. Ions were added to build a physiological condition and neutralize the system, and this helped achieve ionic strength. Further, the system was minimized to remove any steric restraints or inappropriate geometries until the maximum force was reduced below 1000 KJ/mol while preparing the system for subsequent MD simulation. The system further went to an equilibration state in two phases; one is the NVT phase to stabilize the temperature and the next is the NPT phase to stabilize. Finally, the 200 ns production run using the CHARMM36 force field was performed to study the dynamic stability and behavior of the complexes. The simulation trajectories were extracted to study the root mean square deviation (RMSD) and fluctuation (RMSF) and to calculate the number of hydrogen bonds formed during the simulation.

### 4.4. MMPBSA Calculation

We employed the molecular mechanics/Poisson–Boltzmann surface area (MM/PBSA) approach using Gromacs to assess the binding affinities of JE–RdRp and Phaeophyceae-derived molecule complexes. This analysis was conducted on the last 50 nanoseconds of the molecular dynamics (MD) simulation trajectories. The MM/PBSA method combines molecular mechanics energies with solvation energies, derived from the Poisson–Boltzmann equation and surface area calculations, to estimate the free energy of binding of the protein-ligand complexes. This approach is renowned for its effectiveness in providing a detailed understanding of molecular interactions within biological systems. By focusing on the final 50 ns of the MD simulations, we ensured that the system had reached a state of equilibrium, thus allowing for a more accurate and representative calculation of the binding free energies of these complexes [53]. 

### 4.5. Principal Component Analysis (PCA) and Free Energy Landscape (FEL)

PCA was conducted on the MD trajectory data to elucidate the protein-ligand complexes’ dominant motions and identify conformational changes pertinent to ligand binding. The FEL was then constructed from the PCA data to visualize the thermodynamic stability and to map the conformational space accessed by the RdRp–ligand complexes during the simulations. This allowed for identifying the most stable binding conformations and the energy barriers between different conformational states.

## 5. Conclusions

This study used computer-aided drug discovery tools to explore the potential of compounds from brown algae as a therapeutic for the Japanese Encephalitis Virus (JEV). The focus was on inhibiting a key enzyme in JEV’s replication cycle, the RNA-dependent RNA polymerase (RdRp). Four natural compounds from brown algae showed promise in binding to the JEV RdRp. CMNPD16749, CMNPD2606, CMNPD27817, and CMNPD23662 had better binding energies than a reference molecule (galidesivir). Further studies confirmed the precision and stability using the Molecular Dynamics (MD) simulations to understand the behavior and structure of the protein-compound complexes. The PCA and free energy landscape described the confirmational changes and energy transcription. The results suggested that these compounds could inhibit JEV RdRp effectively. In short, this research not only adds to our understanding of marine-derived compounds as potential antiviral agents but also offers a detailed computational analysis of how compounds from brown algae interact with JEV RdRp. The identified compounds could be developed into effective antiviral drugs against JEV and possibly other related viruses. This highlights the importance of combining computational methods with marine biology and natural product chemistry in drug discovery.

## Figures and Tables

**Table 1:** Compound ID in the CMNPD database, compound name, and their marine brown alga source species name.

Compound ID	Compound Name	Compound Source Species Name
CMNPD16749	Sargothunbergol A	*Sargassum thunbergii* [20]
CMNPD2606	Amentadione	*Carpodesmia amentacea* [21]
CMNPD27817	Cystone A	*Cystoseira usneoides* [22]
CMNPD23662	Cystodione C	*Cystoseira usneoides* [23]

**Table 2:** The intermolecular interactions of selected brown alga natural compounds docked with JEV–RdRp.

Serial Number	Complex	H-Bond	Hydrophobic	π–π Stacking/ *π–Cation Interaction	Salt Bridge	Glycine
1.	JEV–RdRp–CMNPD16749	Val^607^, Cys^714^, Arg^742^, Ser^804^	Leu^411^, Ala^413^, Val^514^, Val^607^, Val^608^, Tyr^610^, Cys^714^, Leu^739^, Met^766^, Leu^770^, Tyr^771^, Trp^800^, Ile^802^	--	Lys^463^, Arg^734^, Arg^742^	Gly^412^, Gly^605^
2.	JEV–RdRp–CMNPD2606	Ala^475^, Arg^460^, Val^607^, Ile^802^	Leu^411^, Ala^413^, Val^414^, Ala^475^, Ile^476^, Trp^477^, Val^607^, Val^608^, Tyr^610^, Cys^714^, Trp^800^, Ile^802^	--	--	Gly^412^, Gly^603^, Gly^605^
3.	JEV–RdRp–CMNPD27817	Ala^413^, Arg^474^, Val^607^, Tyr^610^, Arg^742^, Ser^799^, Ile^802^	Ala^410^, Leu^411^, Ala^413^, Val^607^, Val^608^, Tyr^610^, Ala^611^, Cys^714^, Trp^800^, Ile^802^	Lys^463*^	--	Gly^412^
4.	JEV–RdRp–CMNPD23662	Gln^606^, Trp^800^, Ile^802^	Leu^411^, Ala^413^, Val^607^, Tyr^610^, Cys^714^, Trp^800^, Ile^802^	--	--	Gly^412,^ Gly^605^
5.	JEV–RdRp–galidesivir	Pro^464^, Arg^734^, Gly^735^,Glu^738^	Ala^470^, Pro^464^, Pro^712^, Pro^713^, Cys^714^	--	--	Gly^465^, Gly^735^

## Data Availability

The original data presented in the study are included in the article/Supplementary Material; further inquiries can be directed to the corresponding author.

## Supplementary Materials

The following supporting information can be downloaded at: https://www.mdpi.com/article/10.3390/md22020092/s1, Figure S1: 3D and 2D interaction diagram of protein ligand interaction of JEV-RdRp with Genidesivir as reference molecule; Figure S2: RMSD value resulting from JEV-RdRp docked with Genidesivir as reference molecule during molecular dynamics simulation over 200 ns; Figure S3: The protein root mean square fluctuation (P-RMSF) of JEV-RdRp, docked with Genidesivir as reference molecule during 200 ns simulation; Figure S4: Number of hydrogen bonds present in JEV-RdRp docked with Genidesivir as reference molecule during 200 ns simulation; Figure S5: Scatter plot of JEV-RdRp docked with Genidesivir as reference molecule during 200 ns simulation; Figure S6: The free energy landscape of JEV-RdRp docked with Genidesivir as reference molecule; Table S1: Virtual screening results of the top 154 marine fungi compounds; Table S2: MM-PBSA Free Energy Components for selected compounds.
