# Supplementary material for: Marine Brown Algae-Derived Compounds as Potential Inhibitors of Japanese Encephalitis Virus RNA-Dependent RNA Polymerase
Source: Mar Drugs. 2024 Feb 17;22(2):92. doi: 10.3390/md22020092 (PMC10890675; doi:10.3390/md22020092)
Supplement: Supplementary file 1 [file marinedrugs-22-00092-s001.zip › marinedrugs-2850349-supplementary.pdf]

Table S1. Virtual screening results of the top 154 marine fungi compounds

S.N.	Compounds	docking score	S.N.	Compounds	docking score
1.	CMNPD16749	-15.7	78.	CMNPD5356	-7.8
2.	CMNPD2606	-15.2	79.	CMNPD5376	-7.8
3.	CMNPD27817	-14.8	80.	CMNPD5363	-7.8
4.	CMNPD23662	-14.2	81.	CMNPD4683	-7.8
5.	CMNPD14869	-11.6	82.	CMNPD14030	-7.8
6.	CMNPD14874	-11.5	83.	CMNPD21107	-7.8
7.	CMNPD4727	-10.5	84.	CMNPD536	-7.8
8.	CMNPD4727	-10.3	85.	CMNPD7318	-7.8
9.	CMNPD2215	-10.3	86.	CMNPD5903	-7.7
10.	CMNPD2215	-10.1	87.	CMNPD5916	-7.7
11.	CMNPD2209	-10.1	88.	CMNPD6595	-7.7
12.	CMNPD2214	-10	89.	CMNPD533	-7.7
13.	CMNPD13351	-10	90.	CMNPD1801	-7.7
14.	CMNPD30999	-9.7	91.	CMNPD535	-7.7
15.	CMNPD31000	-9.5	92.	CMNPD535	-7.7
16.	CMNPD30998	-9.5	93.	CMNPD27816	-7.6
17.	CMNPD2605	-9.5	94.	CMNPD455	-7.6
18.	CMNPD2208	-9.4	95.	CMNPD23659	-7.6
19.	CMNPD2214	-9.4	96.	CMNPD23663	-7.6
20.	CMNPD2212	-9.2	97.	CMNPD5910	-7.6
21.	CMNPD2212	-8.8	98.	CMNPD26461	-7.6
22.	CMNPD2609	-8.8	99.	CMNPD533	-7.6
23.	CMNPD8816	-8.7	100.	CMNPD5355	-7.6
24.	CMNPD2213	-8.7	101.	CMNPD13329	-7.6
25.	CMNPD23665	-8.7	102.	CMNPD3155	-7.5
26.	CMNPD11763	-8.7	103.	CMNPD416	-7.5
27.	CMNPD8048	-8.6	104.	CMNPD27811	-7.5
28.	CMNPD5368	-8.6	105.	CMNPD27811	-7.5
29.	CMNPD436	-8.6	106.	CMNPD5372	-7.4
30.	CMNPD3177	-8.5	107.	CMNPD489	-7.4
31.	CMNPD8046	-8.5	108.	CMNPD22333	-7.4
32.	CMNPD5916	-8.5	109.	CMNPD18931	-7.4
33.	CMNPD5918	-8.4	110.	CMNPD27816	-7.4
34.	CMNPD8815	-8.4	111.	CMNPD5908	-7.4
35.	CMNPD4706	-8.4	112.	CMNPD9492	-7.4
36.	CMNPD18937	-8.4	113.	CMNPD7317	-7.4
37.	CMNPD1813	-8.4	114.	CMNPD17788	-7.3
38.	CMNPD13326	-8.4	115.	CMNPD23659	-7.3
39.	CMNPD5920	-8.3	116.	CMNPD5355	-7.3
40.	CMNPD5918	-8.3	117.	CMNPD5355	-7.3
41.	CMNPD5923	-8.3	118.	CMNPD5356	-7.3
42.	CMNPD2218	-8.3	119.	CMNPD18936	-7.3
43.	CMNPD447	-8.3	120.	CMNPD22332	-7.3
44.	CMNPD17789	-8.3	121.	CMNPD3175	-7.3
45.	CMNPD5920	-8.3	122.	CMNPD5355	-7.3
46.	CMNPD5919	-8.2	123.	CMNPD5355	-7.3
47.	CMNPD5918	-8.2	124.	CMNPD27817	-7.2
48.	CMNPD18924	-8.2	125.	CMNPD1846	-7.2
49.	CMNPD1803	-8.2	126.	CMNPD17789	-7.2
50.	CMNPD5918	-8.2	127.	CMNPD5355	-7.2
51.	CMNPD5911	-8.2	128.	CMNPD22356	-7.2
52.	CMNPD6604	-8.1	129.	CMNPD17772	-7.2
53.	CMNPD5918	-8.1	130.	CMNPD1846	-7.2
54.	CMNPD5916	-8.1	131.	CMNPD5356	-7.2
55.	CMNPD5919	-8.1	132.	CMNPD5908	-7.2
56.	CMNPD14846	-8.1	133.	CMNPD5908	-7.2
57.	CMNPD4719	-8	134.	CMNPD5355	-7.1
58.	CMNPD5920	-8	135.	CMNPD18933	-7.1
59.	CMNPD5362	-8	136.	CMNPD18936	-7.1
60.	CMNPD5377	-8	137.	CMNPD5356	-7.1
61.	CMNPD5923	-8	138.	CMNPD10217	-7.1
62.	CMNPD5369	-8	139.	CMNPD23657	-7

63.	CMNPD2227	-8	140.	CMNPD22331	-7
64.	CMNPD4717	-8	141.	CMNPD17771	-7
65.	CMNPD17804	-8	142.	CMNPD14006	-7
66.	CMNPD5921	-8	143.	CMNPD5356	-7
67.	CMNPD224	-8	144.	CMNPD5355	-6.9
68.	CMNPD5375	-8	145.	CMNPD7317	-6.9
69.	CMNPD5919	-8	146.	CMNPD5902	-6.9
70.	CMNPD5362	-7.9	147.	CMNPD17769	-6.7
71.	CMNPD5356	-7.9	148.	CMNPD5356	-6.7
72.	CMNPD4685	-7.9	149.	CMNPD14006	-6.6
73.	CMNPD5919	-7.9	150.	CMNPD19967	-6.6
74.	CMNPD5376	-7.9	151.	CMNPD9491	-6.5
75.	CMNPD5919	-7.9	152.	CMNPD17770	-6.4
76.	CMNPD2596	-7.9	153.	CMNPD5373	-6.3
77.	CMNPD533	-7.9	154.	-	-

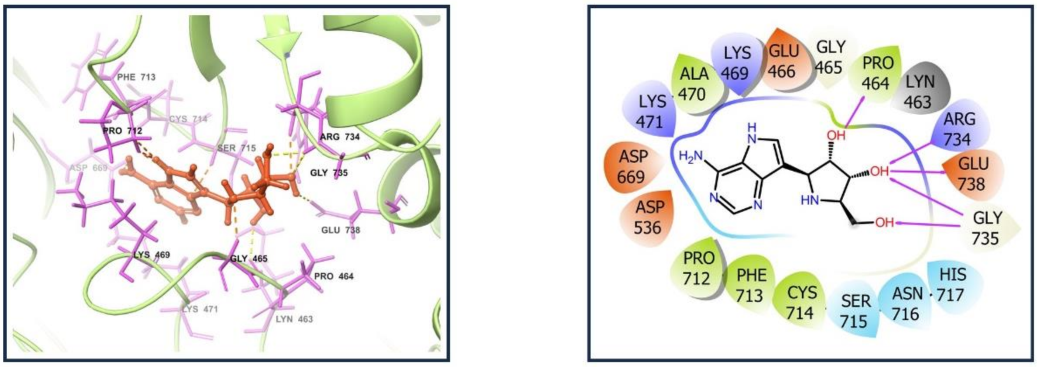

Supplementary Figure S1- 3D and 2D interaction diagram of protein ligand interaction of JEV-RdRp with Genidesivir as reference molecule.

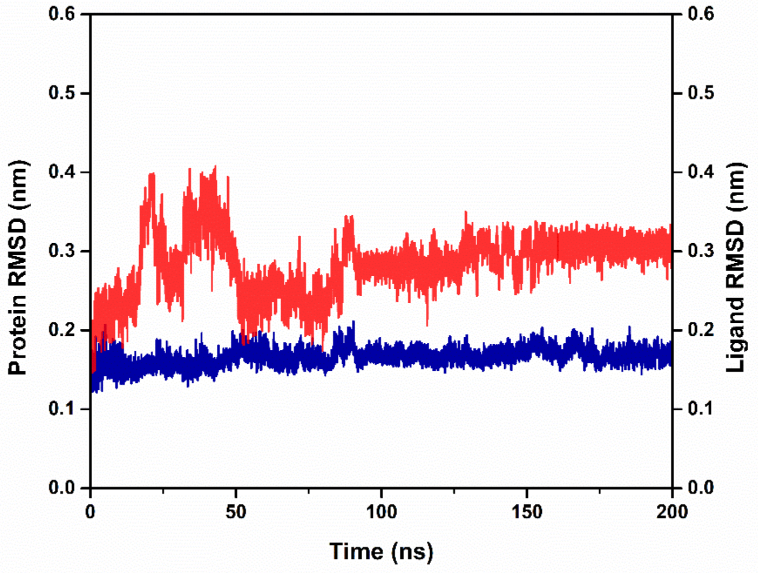

Supplementary Figure S2- RMSD value resulting from JEV-RdRp docked with Genidesivir as reference molecule during molecular dynamics simulation over 200 ns.

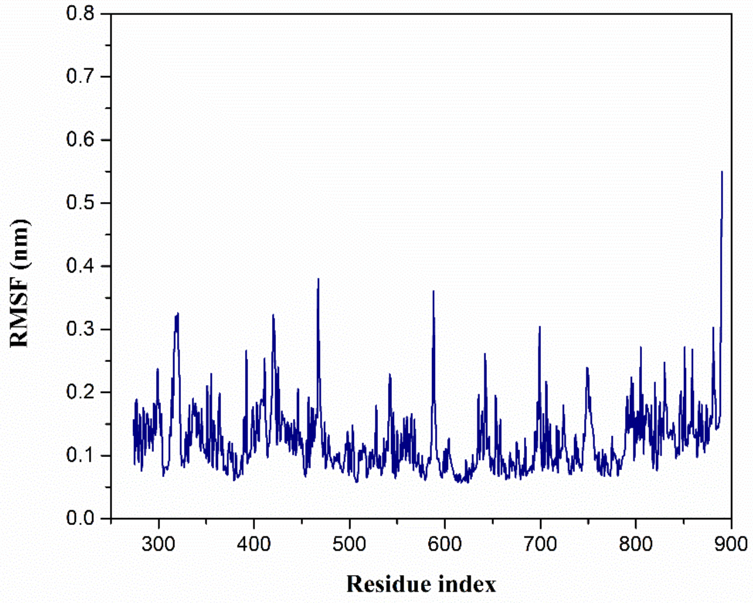

Supplementary Figure S3- The protein root mean square fluctuation (P-RMSF) of JEV-RdRp, docked with Genidesivir as reference molecule during 200ns simulation.

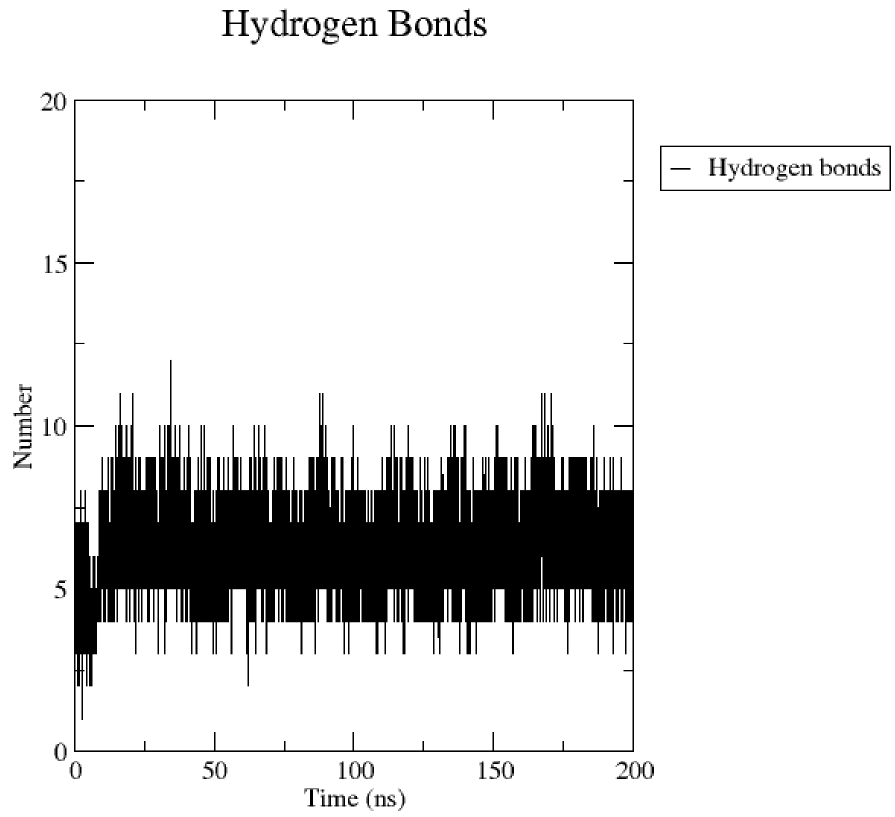

Supplementary Figure S4- Number of hydrogen bonds present in JEV-RdRp docked with Genidesivir as reference molecule during 200ns simulation.

Table S2- MM-PBSA Free Energy Components for selected compounds.

Energy component/ Complexes	JEV-RdRp_CMNPD16749	JEV-RdRp_CMNPD2606	JEV-RdRp_CMNPD27817	JEV-RdRp_CMNPD23662	JEV-RdRp_Galidesivir
ΔVDWAALS	-35.79	-47.3719	-54.76	-36.67	-39.04
ΔEEL	-322.4	-18.0373	-23.26	-32.82	-37.17
ΔEGB	302.4	37.2955	49.92	51.46	49.50
ΔESURF	-7.65	-5.3397	-8.43	-5.51	-6.35

ΔGGAS	-358.18	-65.4092	-78.02	-69.49	-76.21
ΔGSOLV	294.75	31.9557	41.49	45.95	43.15
ΔTOTAL	-63.43	-33.4534	-36.53	-23.54	-33.06

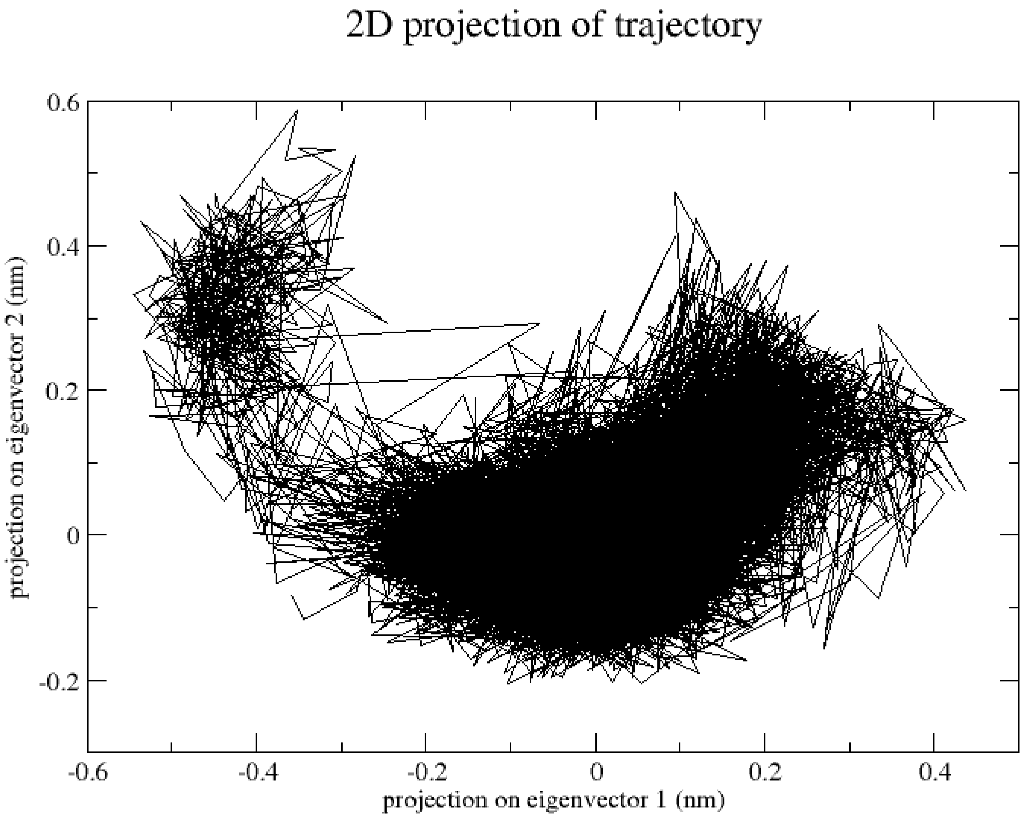

Supplementary Figure S5- Scatter plot of JEV-RdRp docked with Genidesivir as reference molecule during 200ns simulation

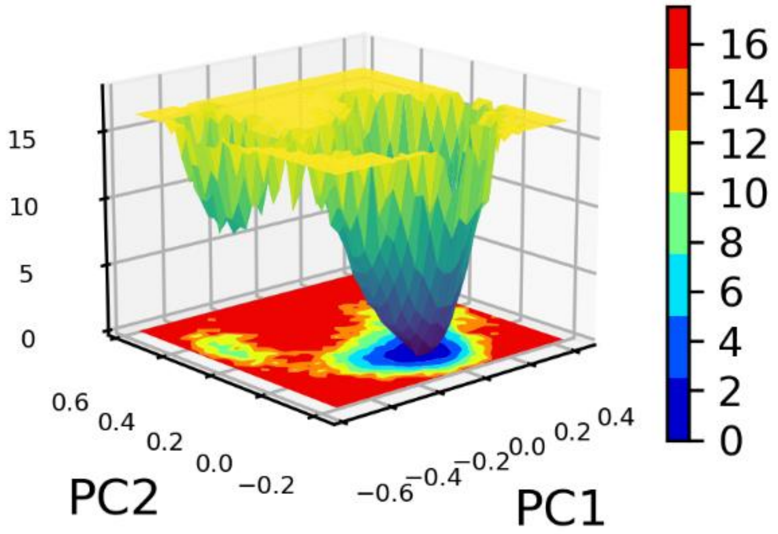

Supplementary Figure S6- The free energy landscape of JEV-RdRp docked with Genidesivir as reference molecule.
